# ASCO Resource-Stratified Guidelines: Methods and Opportunities

**DOI:** 10.1200/JGO.18.00113

**Published:** 2018-08-15

**Authors:** Sana Al-Sukhun, Sarah Temin, Mariana Chavez-MacGregor, Neelima Denduluri, Thomas K. Oliver, Doug Pyle, Manish A. Shah, Julie Gralow

**Affiliations:** **Sana Al-Sukhun,** Jordanian Oncology Society, Amman, Jordan; **Sarah Temin, Thomas K. Oliver,** and **Doug Pyle,** American Society of Clinical Oncology, Alexandria; **Neelima Denduluri,** Virginia Cancer Specialists, Arlington, VA; **Mariana Chavez-MacGregor,** University of Texas MD Anderson Cancer Center, Houston, TX; **Manish A. Shah,** New York-Presbyterian/Weill Cornell Medical Center, New York, NY; and **Julie Gralow,** University of Washington, Seattle, WA.

## Abstract

The objectives of this article are to describe the ASCO Resource-Stratified Guidelines and to provide background within the context of ASCO Guidelines and efforts to address the global cancer burden.

## INTRODUCTION

Cancer has become one of the leading causes of morbidity and mortality worldwide, with a disproportionately rising burden in low- and middle-income countries (LMICs), where 60% to 70% of the world’s total new cancer cases are diagnosed, as well as 70% of cancer deaths.^1-3^ Worldwide, almost 70% of the annual total deaths are caused by noncommunicable diseases, principally from cardiovascular disease, diabetes, chronic respiratory disease, and cancer.^4^ In 2012, nearly 80% of all deaths from noncommunicable diseases occurred in LMICs, with cancer being the leading cause of death among those younger than 70 years.^5,6^ Globally, the economic toll from cancer is 19% higher than from cardiovascular disease, not considering direct medical costs and total number of years lost with subsequent impact on productivity. The latter makes cancer the largest single burden on total economy when compared with HIV and other infectious diseases.^7^

Health authorities, clinicians, policy makers, patients, and families in resource-constrained settings can experience a lack of expert guidance, as well as resources to address the cancer burden. Different international organizations and societies are working together to help improve access to cancer control worldwide.^8^ ASCO, recognizing its role as a leading oncology society, with one third of its 45,000 members from outside the United States/Canada, launched ASCO International on World Cancer Day 2013. ASCO International’s volunteer programs include professional development, education, training, quality improvement, and support for and dissemination of research in LMICs.^9^ “ASCO International connects the global community of cancer care providers through a large and expanding portfolio of international programs.”^10^ ASCO considered the reality that lack of resources (including infrastructure, personnel, and training) can make it difficult for some clinicians/institutions outside the United States to implement ASCO guidelines and thus began publishing Resource-Stratified Guidelines. This article aims to review the background, methods of development, and dissemination of ASCO Resource-Stratified Guidelines.

Effective control of cancer requires integration of effective cancer prevention, early detection, and comprehensive therapeutic and palliative approaches. Because of competing priorities, as well as increased effective options for prevention and treatment, policy makers in LMICs and other resource-constrained settings are facing increasingly difficult decisions regarding budget priorities. Therefore, the difficulty of offering each service optimally is escalating. ASCO recognizes that human resources, poverty, human rights, education, medical system, and cultural and political issues are all influential in local medical and public health practices.

ASCO has long-standing experience with developing evidence-based clinical practice guidelines, with a current portfolio of 85 guidelines, with 25 de novo guidelines and 15 guideline updates in development. ASCO started developing guidelines in 1995. Those guidelines provide detailed and medically sound compilations of updates, insights, advice, and recommendations in the areas of greatest clinical need and uncertainty to ensure that patients get the best-quality medical care. However, they were developed in the context of maximal resources being available and are not likely to be applicable within other resource settings.

ASCO became interested in leveraging the expertise of its international membership and its expertise in clinical practice guideline development to increase guidance on cancer prevention and care around the world by addressing variations in the availability of resources. The guidelines are intended to complement—but not replace—local guidelines, because local guidelines can better reflect local situations, whereas ASCO guidelines take a more globally applicable perspective. The Breast Health Global Initiative (BHGI), of which ASCO was a supporter, developed evidence-based, economically feasible, and culturally appropriate guidelines for nations with limited health care resources to improve breast cancer outcomes.^11^ ASCO has adapted BHGI’s four-tier resource-stratification levels. BHGI resource definitions were later adopted by many oncology societies in an attempt to help physicians deliver the best possible care in limited-resource settings, by efficiently allocating resources. Other groups producing recommendations for resource-stratified settings include the Asian Oncology Summit,^12^ Disease Control Priorities (DCP3),^6^ National Comprehensive Cancer Network,^13^ and WHO,^14^ among others.

ASCO’s International Affairs and Clinical Practice Guidelines Committees proposed a resource-stratified approach using ASCO’s well-known, high-quality guideline development to the ASCO Board of Directors with the goal of creating flexible, resource-stratified methods to practice guidelines, where clinicians and health authorities can identify how existing resources can be optimally applied to improve cancer outcomes, and to provide a bridge for improving cancer care in various health systems. After the Board approved the proposal, ASCO initiated a pilot effort to develop Resource-Stratified Guidelines for prevention of cervical cancer and treatment of women with cervical cancer, a globally high-incidence cancer, and to use the lessons learned from that initiative to inform a structured approach for all common cancers.

The resource-stratified cervical cancer guidelines addressed primary prevention, secondary prevention/screening, work-up, and treatment.^15-17^ Under the leadership of a Resource-Stratified Guidelines Advisory Group, ASCO expanded this suite of products into a planned series of Resource-Stratified Guidelines continuing with palliative care and colorectal cancer, with more topics to be determined over time.^18^ Those topics were suggested and prioritized by the Resource-Stratified Guidelines Advisory Group, which included members from the International Affairs and Clinical Practice Guidelines Committees, representing members from all resource settings.

## METHODS

The target audiences for the guidelines are clinicians, program planners, public health providers, health/public health authorities, policy makers, patients, and caregivers. The ASCO guidelines program began developing Resource-Stratified Guidelines using methods adapted from other established methodologies, including ASCO’s systematic review techniques and consensus methodology, and the ADAPTE methodology.^19,20^ ASCO began developing these guidelines to provide expert guidance for situations in which maximal resources are not available.

### Overview

ASCO sets a high priority on assembling multidisciplinary Expert Panels with members from multiple resource settings. Members represent geographic, northern/southern hemisphere, sex, discipline (and so on) diversity; most members are from (or have extensive experience in) basic- and limited- (indeed, from all four settings) resource settings. All Expert Panels include patient advocates and/or patients and, inspired by BHGI, each Panel meeting starts with the advocate’s presentation. When available, health economists with expertise in these settings are included throughout ASCO’s Resource-Stratified Guidelines initiative. Participation in Expert Panels is not limited to ASCO members, which results in the participation of public health, health economics, and other experts in the ASCO Expert Panels. ASCO has long-established guideline development procedures, including systematic reviews and formal consensus using a modified Delphi strategy and has now used these and other validated methods for some of the recommendations to develop Resource-Stratified Guidelines. These methods have included a modified ADAPTE method, elements of ASCO’s endorsement processes, and, due to the paucity of evidence from some of these settings, formal consensus.^21^

### Systematic Review

All ASCO guidelines begin with a systematic review of the evidence, including a literature search with prespecified elements including inclusion/exclusion criteria. This includes looking for existing guidelines to adapt (part of ADAPTE). Searches are conducted in PubMed and related databases, such as the Guidelines International Network, and relevant Web sites for published high-quality guidelines, especially those published by established developers, preferably professional societies and/or national/international authorities that used high-quality/standardized methods in guideline development (eg, Centers for Disease Control and Prevention, WHO).

### Modified ADAPTE Process

**Fig 1:**
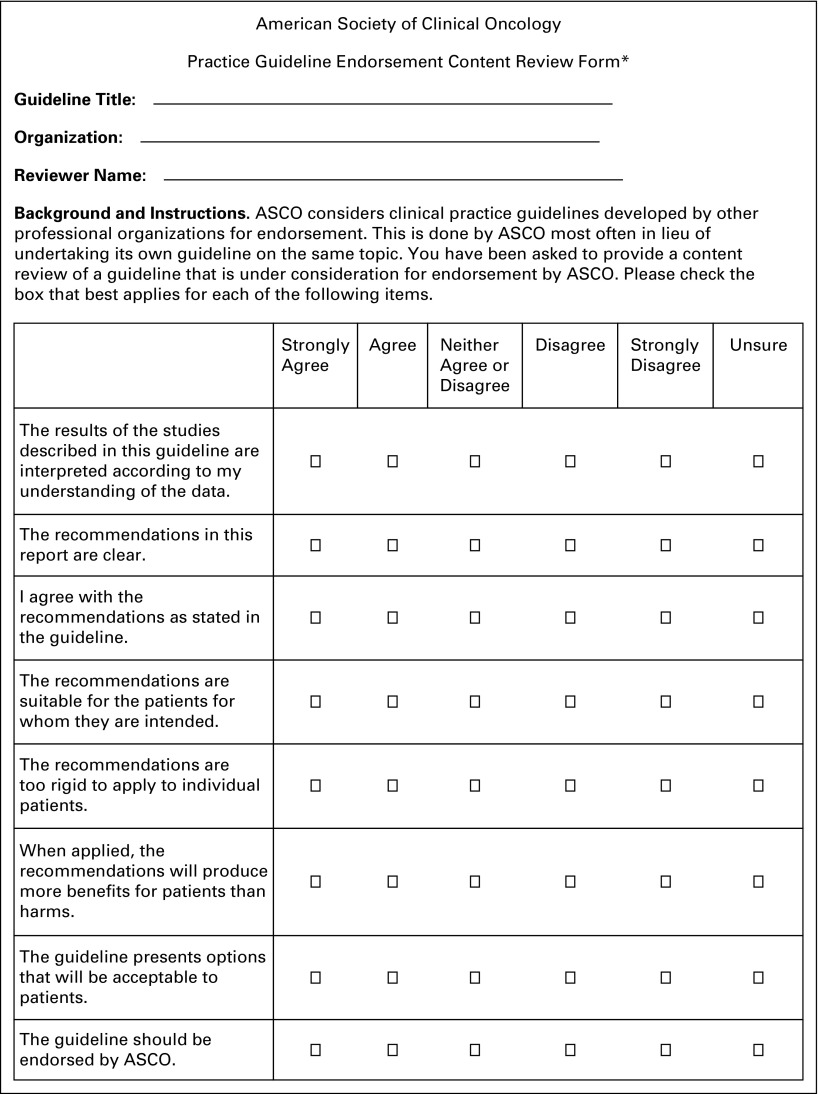
ASCO Guideline Endorsement Content Review Form. (*) This form was adapted from the Cancer Care Ontario Program in Evidence-Based Care Practitioner Feedback instrument. This form was used for ASCO’s Resource-Stratified Guidelines published 2016 to 2018. ASCO may change its Guideline Endorsement Content Review Form in the future; please visit https://www.asco.org/practice-guidelines/quality-guidelines/guidelines-tools-resources/guidelines-development for the most current versions.

If relevant guidelines are identified, the ADAPTE process^20^ involves content review by volunteer experts and methodology review by ASCO staff. One to two Panel members review each guideline for content using the ASCO Endorsement Form (Fig 1) and one to two staff members use the Appraisal of Guidelines for Research and Development II (AGREE II) instrument, Rigor of Development subscale (Table 1)^22,23^ to assess methodological rigor.

**Table 1:**
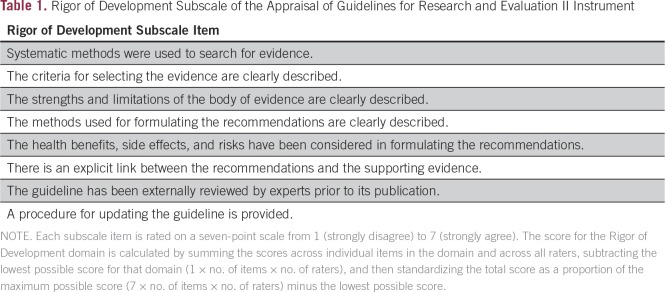
Rigor of Development Subscale of the Appraisal of Guidelines for Research and Evaluation II Instrument

### Recommendations Development

Recommendations are initially developed by the Panel co-chairs and/or Steering Group and then shared with the full Panel or, alternatively, the recommendations are drafted by the full Panel. Primarily done in person, recommendation development uses standard ASCO guideline recommendation syntax. The guideline recommendations are crafted, in part, using the GuideLines Into Decision Support (GLIDES) methodology.^24^ This method helps Guideline Expert Panels systematically develop clear, translatable, and implementable recommendations using natural language, based on the evidence and assessment of its quality, and incorporates distilling the actions involved, identifying implementers (to whom and under what circumstances), and clarifying if and how end users can carry out the actions consistently.

### Formal Consensus

When the Expert Panel deems the available literature insufficient to inform an evidence-based recommendation to guide clinical practice, a formal consensus process is used to develop recommendations, which are considered the best current guidance for practice.^21^ The Panel is then supplemented by additional experts (ie, a Consensus Ratings Panel) to independently rate levels of agreement with the recommendations. The levels of agreement must collectively meet a prespecified threshold (eg, ≥ 75%) that the experts must have rated “agree” or “strongly agree” for each recommendation. The results are reported either in the guideline or its supplement. More details on ASCO methodologies are available in the ASCO Methodology Manual, including a description of formal consensus, systematic review, literature search terms, Appraisal of Guidelines for Research and Development II (AGREE II), and more.

## IMPLEMENTATION

ASCO publishes each Resource-Stratified Guideline in the open access journal, *Journal of Global Oncology* (JGO), and a summary of the guideline in *Journal of Oncology Practice* (JOP). ASCO started JGO to try to address the concerning paucity of research in LMICs. Dr. David Kerr was the founding editor-in-chief and Dr. Gilberto Lopes is the current editor-in-chief. Resource-Stratified Guidelines are also included in ASCO’s guidelines app, and ASCO leverages its social media and other communications mechanisms (eg, Pocket Cards; www.asco.org/practice-guidelines/quality-guidelines/guidelines/guideline-pocket-cards) to disseminate the guidelines. In addition, ASCO University has produced a new, case-based course series designed to provide further essential guideline information to both US- and international-based ASCO members. Resource-Stratified Guidelines are also freely available through ASCO Web sites (www.asco.org, www.cancer.net) and in the app. To assist dissemination in Spanish-speaking areas, ASCO has translated the summaries of the first three Resource-Stratified Guidelines into Spanish.

The guidelines have also been disseminated through the ASCO International programs in LMICs. ASCO’s Cancer Control for Primary Care course trains primary care providers on cancer prevention, cancer screening, and referral systems to oncology specialists; the Guidelines have been shared in support of the cervical cancer training of this course. In a collaboration with Health Volunteers Overseas, ASCO is assisting hospitals in LMICs to improve the quality of care provided to patients with cancer, and the Guidelines have been incorporated into that assistance. The ASCO/Conquer Cancer International Development and Education Award assists early career oncologists in LMICs, and today there are more than 300 recipients of this award in more than 60 countries. The Guidelines have been disseminated to the International Development and Education Award network for further promotion and adoption in their practice settings.

Other groups have endorsed or otherwise recognized ASCO Resource-Stratified Guidelines, including Society for Gynecologic Oncology (endorsement of Treatment guideline), International Gynecologic Cancer Society (endorsement of Secondary Prevention [SP]), American College of Obstetrics and Gynecology (support of Primary Prevention and SP), American Society for Colposcopy and Cervical Pathology (endorsement of SP), and Gynecologic Cancer Intergroup (endorsement of Treatment guideline), and ASCO has worked with these and other groups to disseminate/link to the guidelines. Clinicians in several countries/regions are implementing and/or adapting the guidelines, including in Uganda, Turkey, Lebanon, Central America, and Myanmar. ASCO will continue to work with local oncology societies on disseminating the guidelines.

## DISCUSSION

The dramatic increase in the toll of cancer in LMICs mandates effective interventions at all levels of cancer care. Considering the rising costs of cancer care globally, optimal use of resources to optimize outcome becomes essential. ASCO guidelines offer trustworthy, unbiased, and effective recommended care options that can be used to help plan treatment and management, but do not replace clinician judgement based on the individual circumstances. Resource-Stratified Guidelines give individual clinicians a practical framework for management of each patient, based on the level of health care resources available in the country, region, or practice area where care is being given. Resource-Stratified Guidelines also give policy makers insight into how to plan resource-appropriate cancer control. They are meant to complement local guidelines—not to replace them—and, importantly, provide aspirational and objective criteria to lobby for, or otherwise implement, new resources for existing care and prevention options to reach the next resource stratum. They aim to establish consistent guidance for a minimum primary base of cancer prevention and care and to promote greater resource use and implementation, while realistically accounting for differences in resource levels and health care and public health systems around the world.

## LIMITATIONS

A major limitation of developing Resource-Stratified Guidelines is that the vast complexity and variety of existing health settings make it impossible to provide recommendations using a uniform approach. Rather, the guidelines are intended to provide objectives to help planners reach the next stratum of services while working within the settings of local health situations. We hope that the guidelines can be used as a resource toward local implementation; however, we recognize that much work needs to be done in this area to make implementation a reality.

The development of Resource-Stratified Guidelines is also challenged by limited evidence from the settings where the guidelines are intended to be applied. The limited evidence is partially attributed to a lack of resources needed to conduct research, although this may be changing. Through Conquer Cancer, The ASCO Foundation, ASCO offers International Innovation Grants, which fund research in LMICs. Several of the Innovation Grants have been awarded to principal investigators in LMICs who are conducting research on cervical cancer. The systematic reviews for the guidelines enable the developers to identify gaps in research. Each guideline contains specific suggestions for future research (eg, setting-specific research on a given intervention).

Another challenge is the paucity of implementation of science research in LMICs. In general, research on guideline implementation has found no single effective method, but rather suggests that a combination of methods may be necessary. These methods may include audit and feedback strategies, creating measures, and working with key opinion leaders.^25^ Therefore, ASCO will develop an implementation framework for the purposes of knowledge transfer while continuing its environmental scoping of other such guidelines.

## OPPORTUNITIES

As mentioned earlier, ASCO International organizes live, in-country training courses in LMICs on a range of cancer control topics, and the guidelines have been featured in the “Cancer Control for Primary Care” course. To take this further, ASCO is developing a course on the prevention and management of cervical cancer in LMICs, which will directly draw on the guidelines and facilitate their implementation in these settings. ASCO also anticipates that this course will provide a feedback loop on opportunities and challenges with applying the guidelines under real-world conditions that course participants face. In addition, planning of an evaluation of ASCO Resource-Stratified Guidelines by end users is underway, as well as exploring how to stimulate research to inform updates of the Resource-Stratified Guidelines. ASCO is contemplating piloting efforts to facilitate guideline implementation on a national level in some LMICs. ASCO continues to develop Resource-Stratified Guidelines as it deepens relationships with its international membership and other stakeholders in standing against cancer worldwide. It is the view of ASCO that health care providers and health care system decision makers should be guided by the recommendations for the highest stratum of resources available.

## References

[1] National Cancer Institute Understanding cancer. Cancer statistics.

[2] World Health Organization WHO cancer fact sheet.

[3] International Agency for Research on Cancer World cancer report 2014.

[4] World Health Organization Top 10 causes of death..

[5] World Health Organization Global Health Observatory data repository..

[6] Gelband H, Jha P, Sankaranarayanan R (2015). Disease Control Priorities.

[7] American Cancer Society The global economic cost of cancer..

[8] Al-Sukhun S, de Lima Lopes G, Gospodarowicz M (2017). Global health initiatives of the international oncology community. Am Soc Clin Oncol Educ Book.

[9] Hortobagyi GN, El-Saghir NS, Cufer T (2016). The American Society of Clinical Oncology’s efforts to support global cancer medicine. J Clin Oncol.

[10] American Society of Clinical Oncology ASCO international programs..

[11] Anderson BO, Shyyan R, Eniu A (2006). Breast cancer in limited-resource countries: An overview of the Breast Health Global Initiative 2005 guidelines. Breast.

[12] Lertkhachonsuk AA, Yip CH, Khuhaprema T (2013). Cancer prevention in Asia: Resource-stratified guidelines from the Asian Oncology Summit 2013. Lancet Oncol.

[13] National Comprehensive Cancer Network (2016). NCCN Guidelines® for Global Resource Stratification™: Cervical Cancer.

[14] World Health Organization World Health Organization cancer pages..

[15] Jeronimo J, Castle PE, Temin S (2016). Secondary Prevention of Cervical Cancer: ASCO Resource-Stratified Clinical Practice Guideline. J Glob Oncol.

[16] Arrossi S, Temin S, Garland S (2017). Primary Prevention of Cervical Cancer: American Society of Clinical Oncology Resource-Stratified Guideline. J Glob Oncol.

[17] Chuang LT, Temin S, Camacho R (2016). Management and Care of Women With Invasive Cervical Cancer: American Society of Clinical Oncology Resource-Stratified Clinical Practice Guideline. J Glob Oncol.

[18] Osman H, Shrestha S, Temin S Palliative Care in the Global Setting: ASCO Resource-Stratified Practice Guideline Summary. J Glob Oncol.

[19] Somerfield MR, Bohlke K, Browman GP (2016). Innovations in American Society of Clinical Oncology Practice Guideline Development. J Clin Oncol.

[20] The ADAPTE Collaboration The ADAPTE process: Resource toolkit for guideline adaptation. Version 2.0.

[21] Loblaw DA, Prestrud AA, Somerfield MR (2012). American Society of Clinical Oncology Clinical Practice Guidelines: Formal systematic review-based consensus methodology. J Clin Oncol.

[22] Basch EM, Somerfield MR, Beer TM (2007). American Society of Clinical Oncology endorsement of the Cancer Care Ontario Practice Guideline on nonhormonal therapy for men with metastatic hormone-refractory (castration-resistant) prostate cancer. J Clin Oncol.

[23] Brouwers MC, Kho ME, Browman GP (2010). AGREE II: Advancing guideline development, reporting and evaluation in health care. CMAJ.

[24] Shiffman RN, Michel G, Rosenfeld RM (2012). Building better guidelines with BRIDGE-Wiz: Development and evaluation of a software assistant to promote clarity, transparency, and implementability. J Am Med Inform Assoc.

[25] Pantoja T, Opiyo N, Lewin S

